# Fumonisin-Exposure Impairs Age-Related Ecological Succession of Bacterial Species in Weaned Pig Gut Microbiota

**DOI:** 10.3390/toxins10060230

**Published:** 2018-06-05

**Authors:** Ivan Mateos, Sylvie Combes, Géraldine Pascal, Laurent Cauquil, Céline Barilly, Anne-Marie Cossalter, Joëlle Laffitte, Sara Botti, Philippe Pinton, Isabelle P. Oswald

**Affiliations:** 1 GenPhySE, Université de Toulouse, INRA, INPT, ENVT, 31326 Castanet-Tolosan, France; ivan.mateos-alvarez@inra.fr (I.M.); geraldine.pascal@inra.fr (G.P.); laurent.cauquil@inra.fr (L.C.); celine.barilly@inra.fr (C.B.); 2 Lallemand SAS, 19 rue des Briquetiers, BP 59, 31702 Blagnac CEDEX, France; 3 Toxalim (Research Center in Food Toxicology), Université de Toulouse, INRA, INP-Purpan, ENVT, UPS, 31027 Toulouse, France; anne-marie.cossalter@inra.fr (A.-M.C.); joelle.laffitte@inra.fr (J.L.); philippe.pinton@inra.fr (P.P.); 4 PTP Science Park, via Einstein, loc. Cascina Codazza, 26900 Lodi, Italy; sara.botti@ptp.it

**Keywords:** fumonisin, microbiota, pigs, MiSeq 16S rDNA sequencing

## Abstract

Pigs are highly affected by dietary mycotoxin contamination and particularly by fumonisin. The effects of fumonisin on pig intestinal health are well documented, but little is known regarding its impact on gut microbiota. We investigate the effects of the fumonisin (FB1, 12 mg/kg feed) on the fecal microbiota of piglets (*n* = 6) after 0, 8, 15, 22, and 29 days of exposure. A control group of six piglets received a diet free of FB1. Bacterial community diversity, structure and taxonomic composition were carried out by V3–V4 16S rRNA gene sequencing. Exposure to FB1 decreases the diversity index, and shifts and constrains the structure and the composition of the bacterial community. This takes place as early as after 15 days of exposure and is at a maximum after 22 days of exposure. Compared to control, FB1 alters the ecological succession of fecal microbiota species toward higher levels of *Lactobacillus* and lower levels of the Lachnospiraceae and Veillonellaceae families, and particularly OTUs (Operational Taxonomic Units) of the genera *Mitsuokella*, *Faecalibacterium* and *Roseburia*. In conclusion, FB1 shifts and constrains age-related evolution of microbiota. The direct or indirect contribution of FB1 microbiota alteration in the global host response to FB1 toxicity remains to be investigated.

## 1. Introduction

Food safety is a major issue throughout the world. Therefore, much attention needs to be paid to the possible contamination of food and feed by fungi and the potential risk of mycotoxin production. Mycotoxins are fungal secondary metabolites, potentially hazardous to human and animal health following consumption of contaminated food or feed. These metabolites are very resistant to technological treatments and difficult to remove. Therefore, they can be present in human food and animal feed [1]. Feed materials are often contaminated with fungi and their metabolites, which pose a potential threat to human and animal health [2,3]. Contamination of cereals with mycotoxin is a worldwide problem leading to important economic losses for the agricultural industry. Cereals and soybean are the main components of pig diets and due to the high ingestion of cereals and their sensitivity, pigs are highly impacted by the presence of mycotoxins [4]. The toxicological syndromes caused by ingestion of mycotoxins range from sudden death to reproductive disorders and growth impairment. Consumption of fungal toxins may also decrease resistance to infectious diseases. Pigs are considered to be the farm animals that are the most affected by mycotoxins in general and horses and pigs are the animals that are the most sensitive to fumonisins in particular [5]. Mycotoxin contamination levels in pig feedstuffs are usually not high enough to cause a clinical disease but may result in economic loss through changes in growth, production and immunosuppression [4].

From an intestinal health perspective, the most notorious mycotoxins are fumonisins, especially fumonisin B1 (FB1), and trichothecenes, deoxynivalenol and zearalenone [6]. FB1 is the diester of propane-1,2,3-tricarboxylic acid and 2-amino-12,16-dimethyl-3,5,10,14,15-pentahydro xyeicosane. Pierron et al. [5], reported the toxicity of FB1 and the effects of this mycotoxin on pig intestine, because the intestinal tract is the first barrier and, consequently, the first target of mycotoxins ingested with food. The toxicity of FB1 differs according to several parameters such as the dose, the duration of exposure, the age and the sex of the animal, in additional to nutritional factors. Performances and health are most impacted in young animals and males [7]. The main effects of FB1 are a reduction of feed intake and animal growth, an alteration of the absorptive functionality of the intestine, histological damages on intestinal tissue, an impairment of intestinal barrier functions, a systemic decrease and/or local immune response, as well as lung and liver damages [8].

Gut microbiota plays a key role in physiological, developmental, nutritional and immunological processes of the host, and impacts host health and performance [9]. An appropriate composition of the intestinal microbiota of animals, as well as the quantitative and qualitative stability of that ecosystem, is an essential factor to guarantee animal health. Microbiota provides nutritional and protective functions to animals, by stimulating of host immunity, producing fermentation outputs, and preventing colonization by pathogens [10].

The effect of mycotoxins on the intestinal microbiota is gaining interest [11,12,13]. Nevertheless, the effect of mycotoxins, especially FB1 on the intestinal microbiota is poorly documented [14,15]. The aim of this work was to study the impact of adding FB1 (12 mg/kg) in the diet of piglets on their fecal microbiota during a four-week period of time using high throughput Illumina MiSeq 16S V3–V4 amplicon sequencing. These results complete the data previously published on fumonisin diet contamination host response [8].

## 2. Results

### 2.1. Diversity and Structure Dynamics of the Fecal Bacterial Community

**Figure 1:**
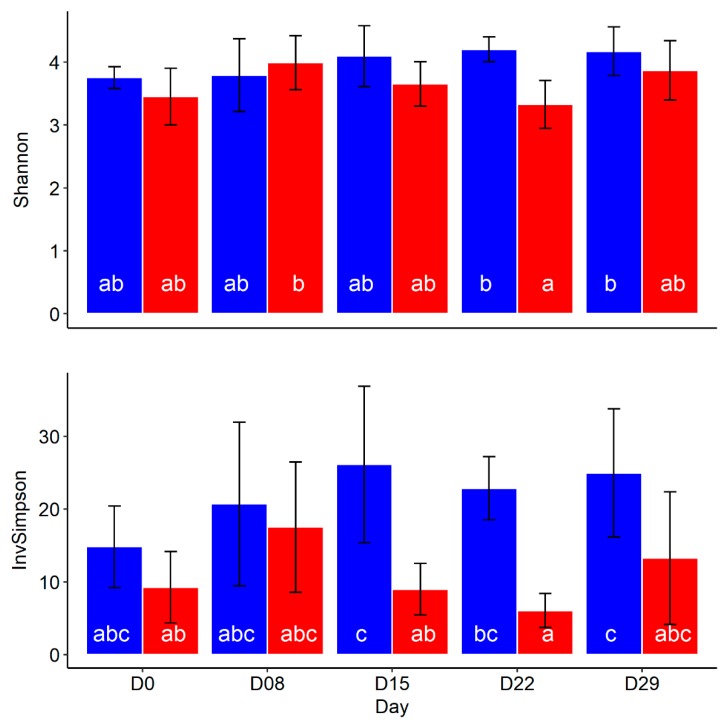
Time effect of dietary FB1 exposure on piglet fecal microbiota Shannon and InvSimpson diversity indexes. In blue, control animals (*n* = 6) and in red, FB1-exposed piglets. (Mean ± SD). LS-means with a common superscript did not differ at *p* = 0.05 level according to linear mixed model analysis of variance.

The effect of fumonisin (FB1) on the gut microbiota of piglets was first assessed on the fecal bacterial community diversity using the Shannon and the InvSimpson indexes (Table 1 and Figure 1). After 4 weeks of exposure to a control diet or a fumonisin-contaminated diet (12 mg/kg) the Shannon and the InvSimpson diversity indexes tended (*p* = 0.057) or was (*p* = 0.003) lower, respectively, in feces from fumonisin-exposed animals compared to those in the Control group. However, for both indexes, significant interaction between age and treatment reveals a differential evolution with age according to groups. In contrast to the Control group where bacterial community diversity is stable over the 4 weeks, the InvSimpson and Shannon indexes decreases after 15 and 22 days of exposure respectively (Figure 1) in fumonisin-exposed piglets.

**Figure 2:**
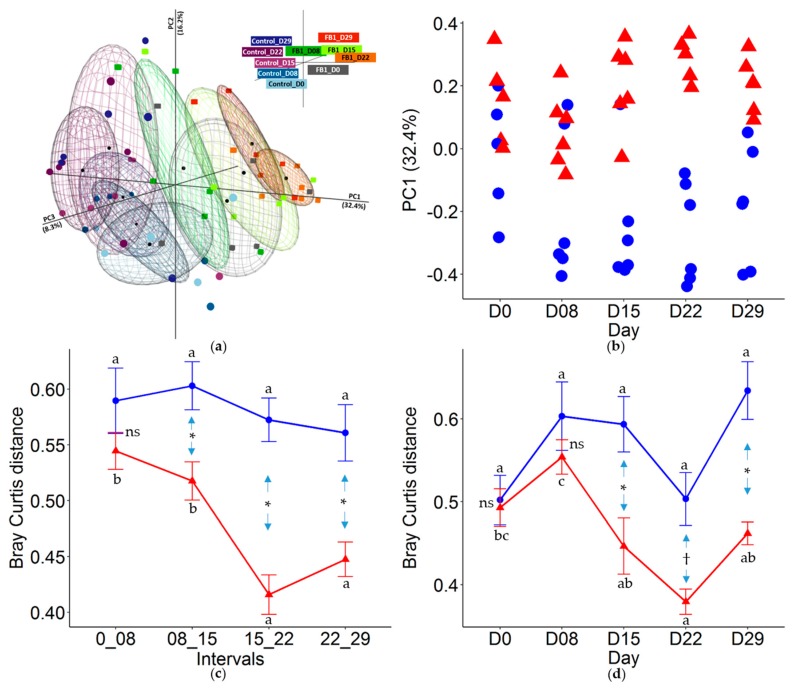
(**a**) Principal coordinate analysis (PCoA) ordination 3D-plot based on the Bray-Curtis distance matrix. Circles and squares are for samples from FB1–exposed and Control animals, respectively. (**b**) PCoA axis1 coordinates plotted against days of treatment. (**c**) Stability between two consecutive age groups calculated from the Bray-Curtis distance for pairwise comparison. (**d**) Age evolution of the individual dispersion within each group using the Bray-Curtis distance. Red triangles: FB1-exposed animals; blue circles: Control animals. * = (*p* < 0.05), † = (*p* < 0.1) and ns = (*p* > 0.1) between groups. a,b = mean with unlike superscripts in a group are significantly different from each other (*p* < 0.05) (*n* = 6 pigs per group, mean ± SEM).

The principal coordinate analysis (PCoA) based on the Bray–Curtis distance (Figure 2a) segregates samples into two groups corresponding to the treatment. Percentages of variance explained by the principal coordinates 1, 2 and 3 are 32.7%, 12.7% and 8.3% respectively. Pairwise ADONIS tests performed on the Bray-Curtis distance matrix (Table S1) indicate that the bacterial community evolves slightly throughout the days of the experiment in both groups of piglets (R^2^-ADONIS < 0.27, *p* < 0.05). When the PCoA axis 1 is plotted against age (Figure 2b) a complete separation of FB1 and Control group can be observed after 22 days of treatment (ADONIS-R^2^ = 0.51, *p* < 0.01, Table S1). To compare the stability of the bacterial community, the distance within two consecutive ages for both groups is calculated (Figure 2c). Except for the 0–8 day interval, the distance between two consecutive ages is lower in the FB1 group than in Control one, suggesting that ingestion of fumonisin contaminated diet hinders the normal rate of age-related evolution of microbiota. Moreover, in the FB1 group, the lowest variation between two consecutive ages is observed for the intervals 15–22 and 22–29 days (*p* < 0.05) suggesting a constraint effect of FB1 on microbiota evolution. Finally, we calculated within-group dispersion, which is the variation of the distance between piglets within an age group (Figure 2d). Individual variations within the group remain unchanged with age in the Control group whereas they sharply decrease in the FB1 group. After 15 days of treatment, the FB1 group exhibits lower within-group distance compared to the Control group. The highest similarity between individuals in the FB1 group is observed at 22 days of age. This result demonstrates that fumonisin exposure exerts a constraint on piglet fecal microbiota evolution that strongly decreases inter-individual variability.

### 2.2. Taxonomic Assignation

**Figure 3:**
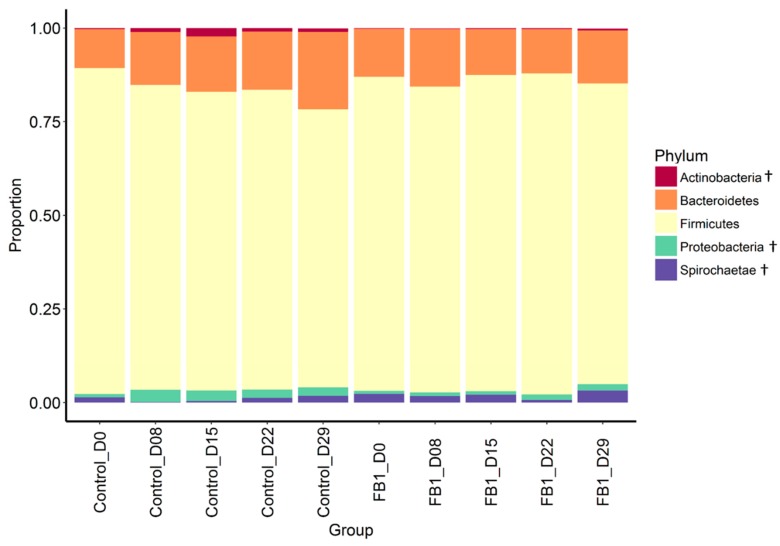
Relative abundance of main phyla in fecal microbiota from Control vs. FB1-exposed pigs. Between treatments: † = *p*-adjusted < 0.10.

Concerning the taxonomic composition of the bacterial community, the Firmicutes phylum is the most abundant one in the microbiota of animals from both groups, with 82% of relative abundance (Figure 3 and Table S2). Bacteroidetes is the second most abundant phylum (14%) followed by Proteobacteria (1.8%) Spirochaetes (1.5%) and Actinobacteria (0.7%). Tenericutes and Fibrobacteres are detected at abundances lower than 0.05% below a quantitative statistical analysis threshold. Actinobacteria and Proteobacteria tend to be observed in higher proportion in Control fecal microbiota than in that of FB1 (1.12% vs. 0.25% and 2.29% vs. 1.18%, respectively, *p*-adjusted < 0.10). Spirochaetes tends to be higher in FB1 than in Control (2.03% vs. 1.01%, *p*-adjusted < 0.10).

**Figure 4:**
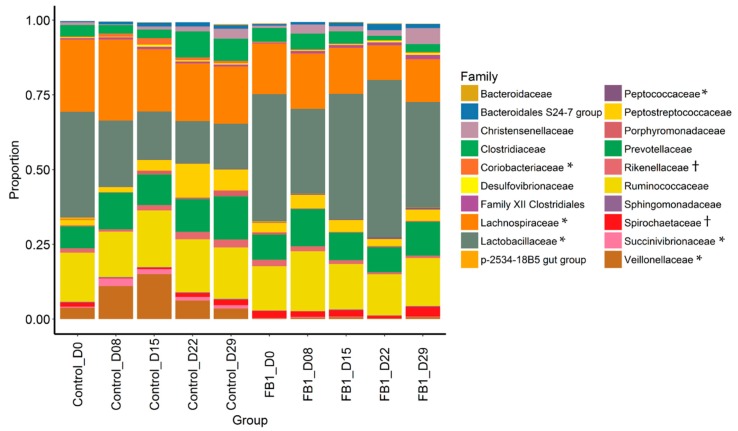
Relative abundance of main families in fecal microbiota from Control vs. FB1-exposed pigs. Between treatments: † = *p* adjusted < 0.10 and * = *p* adjusted < 0.05.

The most abundant families in the samples are Lactobacillaceae, Lachnospiraceae, Ruminococcaceae and Prevotellaceae with more than 10%, Clostridiaceae (4.38%), Peptostreptococcaceae (4.37%) and Veillonellaceae (4.21%) (Figure 4 and Table S3). Compared to Control piglets, fumonisin exposure increases the relative abundance of Lactobacillaceae (*p*-adjusted = 0.031), Peptococcaceae (*p*-adjusted = 0.001), the Bacteroidales RF16 group (*p*-adjusted < 0.001) and the Rickettsiales Incertae Sedis (*p*-adjusted < 0.001) families, and decrases Lachnopiraceae (*p*-adjusted = 0.006), Veillonaceae (*p*-adjusted = 0.005) Eubacteriaceae (*p*-adjusted = 0.009), Succinivibrionaceae (*p*-adjusted = 0.006) and Coriobacteriaceae (*p*-adjusted = 0.031) in fecal microbiota. Among the most abundant families, there is an effect of day on the proportion of Lachnospiraceae that decreases with time, respectively, whereas Veillonaceae increases until day 15 and return to initial values afterward in control group (Figure S1, *p*-adjusted < 0.05).

**Figure 5:**
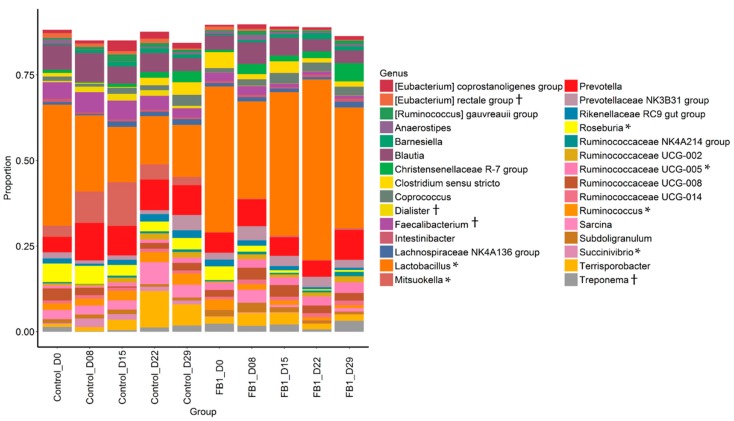
Relative abundance of main genera in fecal microbiota from Control vs. FB1-exposed pigs. Between treatments: † = *p* adjusted < 0.10 and * = *p* adjusted < 0.05.

The 10 most abundant genera (Figure 5) are *Lactobacillus* with (30.3%), *Prevotella* (7.5%), *Blautia* (5.3%), *Terrisporobacter* (3.5%), *Mitsuokella* (3.3%), *Faecalibacterium* (3.0%), *Roseburia* (2.7%), the Prevotellaceae NK3B31 group (2.4%), *Sarcina* (2.4%) and Ruminococcaceae UCG-008 (2.3%), respectively. Out of the 20 genera whose proportions are affected by FB1 exposure (*p*-adjusted < 0.05; Table S4), *Lactobacillus* and Ruminococcaceae UCG-005 are increased while *Mitsuokella*, *Succinivibrio*, *Roseburia* and *Ruminococcus* proportions are depressed (*p* < 0.05).

### 2.3. OTU Differential Abundance

**Figure 6:**
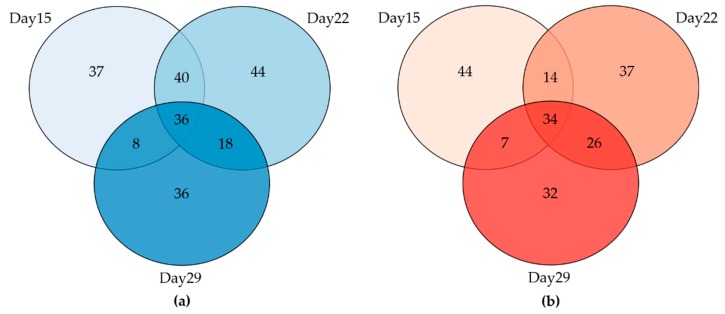
Venn diagram of the number of Operational Taxonomic Units (OTUs) with (**a**) differential lower abundance and (**b**) higher abundance after 15, 22 and 29 days of FB1exposure compared to control.

**Figure 7:**
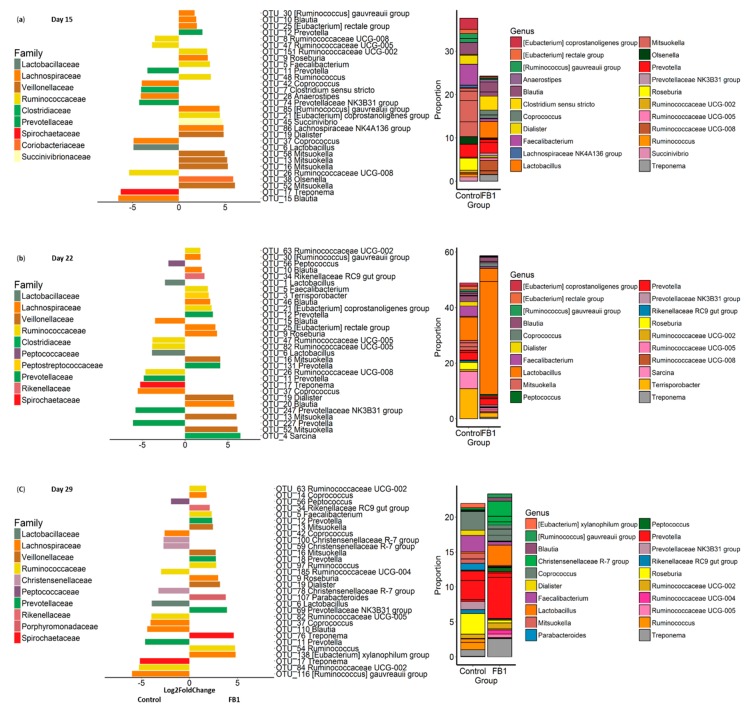
Differential abundance of the 30 most abundant OTUs, (**a**) for day 15, (**b**) for day 22 and (**c**) for day 29. Left panel: log2Fold change abundance, bar color of each OTU are given according to their family assignation, right panel: stacked barplot of the differential abundant OTUs. Each box represent one OTU abundance and color of each OTU are given according to their genus assignation.

To further investigate the FB1-related shift effect on the fecal bacterial community structure, we explored the differential abundance at the OTU (Operational Taxonomic Unit) level. Of the 765 OTUs detected, in the three last days of sampling, 220, 249 and 197 OTUs were differentially abundant between feces from Control and fumonisin-exposed animals (*p* < 0.05, Figure 6). A total of 70 differential abundant OTUs are common to the three days of sampling. Among the 30 most abundant OTUs (Figure 7), consecutive to FB1 exposure, the relative abundance of three of them (OTUs 6, 11 and 17 assigned to the genera *Lactobacillus*, *Prevotella* and *Treponema*, respectively) was increased, whereas the relative abundance of six others was decreased (OTUs 5, 9 12, 13, 16 and 19, assigned to the genera *Faecalibacterium*, *Roseburia*, *Prevotella*, *Mitsuokella* and *Dialister*, respectively). On day 22, the OTU 1 assigned to the *Lactobacillus* genus, reached more than 40% of abundance in FB1 samples (Figure 7b). Thus, the FB1-related shift effect on the fecal bacterial community is mainly explained by the significant abundance changes in the major OTUs that make up fecal bacterial community and that leads to the installation of some dominant OTUs which at least include the two main OTUs assigned to the *Lactobacillus* genera.

In total, the taxonomic composition analysis of the fecal bacterial community reveals that FB1 alters the ecological succession of fecal microbiota species toward higher levels of *Lactobacillus* and lower levels of the Lachnospiraceae and Veillonellaceae families and, particularly OTUs of genera *Roseburia*, *Mitsuokella* and *Faecalibacterium*.

## 3. Discussion

In spite of the economic and health impact of fumonisin diet contamination on livestock and particularly in pigs, to our knowledge, this study is the first to investigate the dynamic effect of FB1 exposure in feed on fecal microbiota in young weaned pigs. In a companion paper, the detrimental effect of FB1 on the same animals was demonstrated, decreasing the growth rate, increasing the ratio Sa/So (which is a marker of the exposure to this mycotoxin in the liver) and decreasing villi length [8]. In the present study, we clearly demonstrate that FB1 exposure in feed impairs age-related evolution of gut microbiota.

In agreement with the literature [16,17,18,19,20], Firmicutes and Bacteroidetes phyla represent more than 90% of the bacterial community OTUs. The bacterial community is dominated by the Lactobacillaceae, Lachnospiraceae, Ruminococcaceae and Prevotellaceae families and *Lactobacillus*, *Prevotella* and *Blautia* genera. Over the time of the experiment (from 35 to 57 days of age), Lachnospiraceae decreased corresponding mainly to a decrease in *Blautia* and *Roseburia* genera. Over the 4 weeks of the experiment, the diversity indexes were stable in the Control group and the structure of the community evolved slightly with high inter-individual variations. Indeed, major changes in diversity occur at birth when the colonization process takes place in neonates in contact with a microbial metacommunity provided by the mother during and after the passage through the birth canal and the surrounding environment [21]. The gut microbiota then undergoes a progressive species age-related succession [17,19,22] driven by both extrinsic factors and intrinsic factors [23]. The extrinsic factors concern surrounding conditions such as housing [24,25], congener proximity [26] and nutritional factors that act throughout the development of the animal. The most impacting one is related to the feed transition from milk to solid feed [19]. The intrinsic factors are those related to the host physiological state, the qualitative and quantitative availability of endogenous nutrients, the motility of the intestinal tract, bile salts and other endogenous secretions, immune tolerance and host-microbiota interactions through PRR and PAMPs (Pattern-Recognition Receptors and Pathogen Associated Molecular Pattern) [27]. All these extrinsic and intrinsic factors shape each individual gut microbial pattern, explaining the inter and intra-individual variability of gut microbiota.

Studies on mycotoxin actions on gut microbiota are rare [11,13] and they mainly concern the effect of deoxynivalenol [12,28,29,30] and aflatoxins [31,32]. After two weeks of dietary exposure to FB1, the diversity of the fecal bacterial community decreases. Concomitantly, a shift in the structure together with a decrease in the rate of the evolution of the bacterial community is observed. In agreement, exposure to FB1 for 29 days in 11 week-old specific pathogen-free pigs modifies the CE-SCCP (Capillary Electrophoresis Single Strand Conformation Polymorphism) bacterial community profiles [14]. Additionally, exposure to FB1 constrains the bacterial community evolution, driving it in a new extremely homogenous equilibrium with low inter-individual variations. Altogether, these results indicate the establishment of some dominant species in the fecal bacterial community that adapt to FB1-related conditions. Considering taxonomic composition of major genera, exposure to FB1 leads to a sharp increase in *Lactobacillus* and Ruminococcaceae UCG-005 and a decrease in *Mitsuokella*, *Roseburia*, *Ruminococcus* and *Succinivibrio*. Several hypothesis might explain solely or in combination these microbiota modifications following FB1 exposure: a direct effect of FB1 on gut microbiota, and/or a direct or an indirect host mediated effect.

One hypothesis to explain the constraint effect of FB1 on piglet fecal microbiota would be an intrinsic antimicrobial effect of mycotoxin. The addition of FB1 to *in vitro* incubation of cecal chime decreased the anaerobic bacteria, whereas *Lactobacillus* and total bacteria increased [15]. In contrast, 20 years ago, Becker et al. [33] used culture techniques and observed no inhibition of bacterial growth including *Lactobacillus acidophilus*, *Lactobacillus johnsoni*, *Lactobacillus plantarum* and *Lactobacillus reuteri*. Finally, two independent studies [34,35] found no antibiotic effect of FB1, thus excluding an antimicrobial effect to explain a fumonisin-related shaping action on gut microbiota.

Considering bacterial community structure evolution and inter individual dispersion criteria and the number of OTUs found to be differentially abundant, the greatest effect of fumonisin on fecal microbiota seems to occur after 22 days of exposure in the diet, whereas at 29 days of exposure, this effect seems to be alleviated, although taxa relative abundance has not yet recovered to levels similar to those observed in the Control group. It may be speculated that the bacterial community might evolve toward a new equilibrium adapted to the exposure to fumonisin. In this latter perspective, microorganisms might metabolize this mycotoxin. Although, 20 years ago, under culture conditions, there was no indication that fumonisin was metabolized by intestinal bacteria [33], it is now admitted that some bacteria isolated from soil and plants are known to degrade and thereby detoxify fumonisins [36]. It was recently reported in pigs that an oral single dose exposure of fumonisin leads to a 47% fumonisin degradation into their partially hydrolyzed forms in the gastro-intestinal tract, thus indicating the ability of the microbiota to hydrolyze fumonisin [37]. The capacity to degrade or remove FB1 *in vitro* was emphasized for *Lactobacillus brevis*, *L*. *plantarum* *L*. *pentosus* and some yeasts [38,39]. The mechanism of action of *Lactobacillus* to remove FB1 was related to a process of physical adsorption involving various components of cell wall [39]. Peptidoglycans was the main binding sites, and its structural integrity was necessary. Therefore, the high proportion of *Lactobacillus* in our work might result from a competitive advantage linked to their ability to metabolize FB1.

In addition to a direct effect on gut microbiota, FB1 might shape microbial composition through its well-known actions on the host. In pigs, exposure to mycotoxins reduces feed intake [1]. Reducing feed intake has been shown to affect both functioning and composition of microbiota [40]. Indeed, reduction of nutrient supply decreases short-chained fatty acid concentration which in turn affect the microbiota composition. In pigs, a favorable effect of feed restriction was observed on *Lactobacillus* abundance [24]. Although, after oral exposure, FB1 is poorly absorbed in the intestine and feces is the main excretory route [37], it induces abdominal pain and diarrhea [1]. In piglets in the present study, it reduced villi length [8]. Impairment of the intestinal barrier function [41] has also been reported. In the jejunum and the ileum of piglets fed deoxynivalenol alone or with fumonisin, the number of goblet cells that synthesize and secrete mucins decreased significantly [42]. Mucins can be an important factor in the shaping of the bacterial community since mucins provide attachment sites and are an endogenous carbon and energy source for intestinal bacteria [43]. Altogether, the reduced feed intake of host and alteration of its intestinal barrier function following exposure to FB1 might modify host control of its symbiotic microbial community.

## 4. Conclusions

The present study investigated the effects of a 4-week dietary exposure to fumonisin on piglet fecal microbiota. We demonstrate that dietary exposure to fumonisin in pigs hinders age-related dynamics of fecal microbiota. Fumonisin decreases the diversity, and shifts and constrains the structure and taxonomic composition of the fecal bacterial community as early as after 15 days of exposure and is at a maximum at 22 days. Dietary exposure to fumonisin promotes the installation of an ecological dominance since it decreases diversity and increases the abundance of *Lactobacillus* at the expense of the abundance of Lachnospiraceae groups, *Roseburia* and *Mitsuokella* genera. The action mechanisms of fumonisin that drive microbiota to this new equilibrium are not known and need further investigations.

## 5. Materials and Methods

### 5.1. Animals, Housing and Experimental Design

This work was done at the same time and with the same animals as the experiment made by Régnier et al. [8]. The study was carried out with twelve 7-week-old weaned castrated male pigs. Animals were obtained from a local farm (Gaec de Calvignac, St. Vincent d’Autejac, France). They were individually identified and divided into two groups and were acclimatized for one week in the animal facility of the INRA ToxAlim Unit (Toulouse, France) prior to being used in experimental protocols. The two groups of six animals, were housed in a separate block of the housing unit with free access to feed and water. Pigs were randomly distributed within pens in order to avoid the effect of the lineage (11.8 ± 1.0 kg and 13.9 ± 1.0 kg, *p* < 0.05, for FB1 and control pigs respectively). Pigs were examined daily for body temperature and feces aspect. No morbidity or mortality was recorded during the study. Room temperature and air velocity were automatically controlled, and pens were cleaned daily. The experiments were carried out in accordance with European Guidelines for the Care and Use of Animals for Research Purposes (accreditation number APAFIS#5917-2016070116429578 v3).

### 5.2. Experimental Diet, Growth Rate and Sample Collection

Diets were formulated as already described [44]. The detailed composition is provided in Table S5. A control diet, and a diet supplemented with fumonisin enriched extract were prepared [8]. Fumonisin extract was obtained from Dr Bailly at the Veterinary School of Toulouse. In brief, *F. verticilliodes* strain NRRL 34281 was cultured on maize grains for 4 weeks at 25 °C. After incubation culture material was dryed and grounded into powder and fumonisin extract content was determined by HPLC/MS-MS. Required quantity of powder was then included in the premix before its inclusion in the final diet. After an acclimation week, pigs were fed with the control or the experimentally contaminated diet (10.2 mg FB1 + 2.5 mg FB2 + 1.5 mg FB3/kg), which constituted day 0 of the trial. Because deoxynivalenol and zearalenone were naturally present in the cereals used, this results in concentrations of 0.12 and 0.015 mg/kg feed, respectively. All other mycotoxins, including aflatoxins, T-2 toxin, HT-2 toxin, and ochratoxin A, were below the limits of detection. Mycotoxins were analyzed in the final diet in the Laboca laboratory (Ploufragan, France) with a LC-MS/MS method described [44].

### 5.3. DNA Extraction and PCR

Total genomic DNA was extracted from 0.5 g of fecal sample, combining a mechanical lysis with a TissueLyser II instrument (Qiagen, Hilden, Germany) and the Quick-DNA™ Fecal/Soil Microbe 96 Kit Zymo Research, Irvine, CA, USA), according to the manufacturer’s instructions [45]. The quality and quantity of DNA extracts were checked using a NanoDrop ND-1000 spectrophotometer (NanoDrop Technologies, Wilmington, DE, USA).

The V3–V4 regions of 16S rRNA genes of samples were amplified from purified genomic DNA with the primers F343 (5′-CTTTCCCTACACGACGCTCTTCCGATCTTACGGRAGGCAGCAG-3′; [46]) and reverse R784 (5′-GGAGTTCAGACGTGTGCTCTTCCGATCTTACCAGGGTATCTAATC CT-3′; [47]). The PCR was carried out with an annealing temperature of 65 °C for 30 amplification cycles. Since MiSeq sequencing machine enables paired 250-bp reads, the ends of each read are overlapped and can be stitched together to generate high-quality, reads of the entire V3 and V4 region in a single run 412 ± 11 nucleotides. At the Genomic and Transcriptomic Platform (INRA, Toulouse, France) single multiplexing was performed using 6 bp index sequences, which were added to R784 during a second PCR with 12 cycles. The resulting PCR products were purified and loaded onto the Illumina MiSeq cartridge (Illumina, San Diego, CA, USA) according to the manufacturer’s instructions. Each pair-end sequence was assigned to its sample with the help of the previously integrated index. Sequencing reads were deposited in the National Center for Biotechnology Information Sequence Read Archive (NCBI SRA; SRP139897).

### 5.4. Sequence Analysis

A total of 1,810,933 16S ribosomal DNA amplicon sequences were sorted based on their respective barcodes, representing the 59 fecal samples. Using FROGS [48], in keeping with the SOP, sequences were filtered by removing sequences that did not match both proximal PCR primer sequences (no mismatch allowed), erroneous sequencing length (<400 or >500 nucleotides), with at least one ambiguous base. Chimeric DNA sequences were detected using VSEARCH and removed. Reads were clustered into OTUs using SWARM [49]. OTU taxonomic assignment was performed using the BLAST algorithm against the SILVA SSU Ref NR 128 database [50]. A phyloseq R package [51] object was generated to perform further statistical analysis.

### 5.5. Statistical Analyses

All statistical analyses were carried out using R software, version 3.4.2 [52] in RStudio software, version 1.1.383 [53]. Shannon and InvSimpson diversity indexes were calculated and the structure of the bacterial community was investigated after calculation of a Bray-Curtis distance matrix that was plot using a Principal Coordinate Analysis, after matrix rarefaction normalization. Using a pairwise Bray Curtis distance calculation, bacterial community stability and inter-individual variability within a group were evaluated between two consecutive day group using the principle of moving window analysis and within each day group respectively [54]. To check group differences an ADONIS pairwise test with the Bray-Curtis distance was carried out. The differential abundance analysis for sequence count data between groups was performed using the DESeq package [55]. Venn diagrams were obtained with the jvenn plug-in [56]. The linear model used had treatment, time and the interaction treatment x time as fixed effects and animal as the random effect.

## Figures and Tables

**Table 1:** Effect of FB1 exposure in the diet on piglet fecal microbiota Shannon and InvSimpson diversity indexes.

Diversity Index	Treatment			*p* Value		
	Control	FB1	SEM	Group	Day	Group × Day
Shannon	4.00	3.67	0.062	0.057	0.067	0.010
InvSimpson	21.9	11.0	1.28	0.003	0.052	0.037

## Supplementary Materials

The following are available online at http://www.mdpi.com/2072-6651/10/6/230/s1, Table S1. Pairwise ADONIS tests between treatments for each day of sampling. Table S2. Phylum relative abundance (%) in fecal microbiota from Control and FB1-exposed piglets. Table S3. Percentage (%) of main bacterial families in fecal microbiota from Control vs. FB1-exposed pigs. Table S4. Percentage (%) of main genera in fecal microbiota from Control vs. FB1-exposed pigs. Table S5. Diet composition in percentage (%). Figure S1 Relative abundance of main bacterial families in fecal microbiota from Control (blue) vs. FB1-exposed pigs (red). Figure S2. Relative abundance of main bacterial genera in fecal microbiota from Control (blue) vs. FB1-exposed pigs (red).
