# Supplementary material for: Fumonisin-Exposure Impairs Age-Related Ecological Succession of Bacterial Species in Weaned Pig Gut Microbiota
Source: Toxins (Basel). 2018 Jun 5;10(6):230. doi: 10.3390/toxins10060230 (PMC6024561; doi:10.3390/toxins10060230)
Supplement: Supplementary file 1 [file toxins-10-00230-s001.pdf]

Supplementary Materials: Fumonisin-Exposure Impairs Age-Related Ecological Succession of Bacterial Species in Weaned Pig Gut Microbiota

Ivan Mateos, Sylvie Combes, Géraldine Pascal, Laurent Cauquil, Céline Barilly, Anne-Marie Cossalter, Joëlle Laffitte, Sara Botti, Philippe Pinton and Isabelle P. Oswald

Table S1. Pairwise ADONIS tests between treatments for each day of sampling. * *p*-adjusted values < 0.05 and † *p*-adjusted-values < 0.10, "false discovery rate" test.

R2	Control D0	Control D8	Control D15	Control D22	Control D29
**Control D0**					
**Control D8**	NS				
**Control D15**	0.235 *	NS			
**Control D22**	0.269 *	0.169 †	NS		
**Control D29**	0.216 *	0.167 †	NS	NS	
**FB1 D0**	0.243 *				
**FB1 D8**		0.229 *			
**FB1 D15**			0.356 *		
**FB1 D22**				0.506 *	
**FB1 D29**					0.297 *

R2 Adonis	<0.25	0.25-0.30	0.30-0.50	>0.50

High R2-ADONIS indicates well separated groups

	**FB1 D0**	**FB1 D8**	**FB1 D15**	**FB1 D22**	**FB1 D29**
**FB1 D8**	NS				
**FB1 D15**	NS	NS			
**FB1 D22**	0.180 *	0.234 *	NS		
**FB1 D29**	0.216 *	0.163 †	NS	0.159 †	

Table S2. Phylum relative abundance (%) in fecal microbiota from Control and FB1 exposed piglets.

Phylum	Treatment[1]			*p*-value			*p*-adjusted[2]	
	Control	FB1	SEM	Group	Date	Group x Date	Group	Date
Actinobacteria	1.12	0.25	0.154	0.012	0.060	0.124	0.059	0.075
Bacteroidetes	15.10	13.30	0.713	0.462	0.125	0.327	0.462	0.125
Firmicutes	80.48	83.17	0.887	0.125	0.054	0.352	0.157	0.075
Proteobacteria	2.29	1.18	0.188	0.047	0.010	0.172	0.079	0.025
Spirochaetae	1.01	2.03	0.254	0.031	0.009	0.108	0.078	0.025

[1] FB1 pigs were fed with a diet supplemented with 12ppm/kg FB1 for 29 days; Control pigs received the same diet without any supplementation. Fecal sampling took place weekly.

[2] Values obtained with "false discovery rate" test.

Table S3. Percentage (%) of main bacterial families in fecal microbiota from Control vs FB1-exposed pigs.

Family	Treatment			*p* value			*p* adjusted	
	Control	FB1	SEM	Group	Date	Group x Date	Group	Date
**Firmicutes**								
Christensenellaceae	1.49	2.55	0.361	0.121	0.001	0.003	0.218	0.006
Clostridiaceae	5.09	3.60	0.694	0.371	0.177	0.000	0.471	0.208
Erysipelotrichaceae	0.07	0.05	0.014	0.019	0.005	0.001	0.051	0.017
Eubacteriaceae	0.11	0.00	0.016	0.002	0.096	0.405	0.009	0.137
Family XIII Clostridiales	0.71	0.92	0.074	0.387	0.008	0.028	0.471	0.024
Lachnospiraceae	22.23	15.33	0.771	0.001	0.000	0.685	0.006	0.001
Lactobacillaceae	20.25	40.20	2.468	0.010	0.051	0.101	0.031	0.084
Peptococcaceae	0.14	0.37	0.031	0.000	0.002	0.005	0.001	0.008
Peptostreptococcaceae	5.08	3.57	0.596	0.419	0.004	0.001	0.471	0.016
Ruminococcaceae	17.09	15.97	0.565	0.395	0.868	0.066	0.471	0.902
Streptococcaceae	0.11	0.07	0.048	0.657	0.053	0.344	0.657	0.084
Veillonellaceae	7.94	0.50	0.920	0.001	0.016	0.029	0.005	0.036
**Bacteroidetes**								
Bacteroidaceae	0.10	0.13	0.034	0.068	0.109	0.605	0.131	0.148
Bacteroidales RF16 group	0.02	0.19	0.024	0.000	0.975	0.011	0.000	0.975
Bacteroidales S24-7 group	1.06	1.23	0.097	0.414	0.000	0.653	0.471	0.001
p-2534-18B5 gut group	0.15	0.11	0.064	0.138	0.013	0.326	0.233	0.032
Porphyromonadaceae	0.81	0.32	0.132	0.166	0.039	0.023	0.263	0.076
Prevotellaceae	11.11	10.07	0.623	0.623	0.046	0.923	0.657	0.083
Rikenellaceae	1.81	1.24	0.121	0.024	0.121	0.000	0.054	0.156
**Proteobacteria**								
Campylobacteraceae	0.08	0.12	0.022	0.397	0.264	0.103	0.471	0.296
Desulfovibrionaceae	0.37	0.35	0.040	0.639	0.001	0.012	0.657	0.007
Rickettsiales Incertae Sedis	0.00	0.11	0.016	0.000	0.001	0.001	0.000	0.005
Sphingomonadaceae	0.26	0.22	0.018	0.325	0.156	0.593	0.471	0.191
Succinivibrionaceae	1.42	0.13	0.177	0.001	0.018	0.434	0.006	0.038
Syntrophaceae	0.06	0.04	0.009	0.021	0.320	0.084	0.051	0.346
**Actinobacteria**								
Coriobacteriaceae	1.08	0.22	0.153	0.010	0.056	0.160	0.031	0.084
**Spirochaetace**								
Spirochaetaceae	1.01	2.03	0.254	0.031	0.009	0.108	0.064	0.024

[1] FB1 pigs were fed with a diet supplemented with 12ppm/kg FB1 for 29 days; Control pigs were fed the same diet without any supplementation. Fecal sampling took place weekly.

[2] Values obtained with "false discovery rate" test.

Table S4. Percentage (%) of main genera in fecal microbiota from Control vs FB1-exposed pigs. Genus are sorted according to their *p*-adjusted value of group effect.

**Genus**	**Treatment**			***p*-value**			***p*-adjusted**	
	**Control**	**FB1**	**SEM**	**Group**	**Date**	**Group x Date**	**Group**	**Date**
[Bacteroides] pectinophilus group	0.16	0.00	0.036	0.000	0.406	0.341	0.000	0.465
Candidatus Hepatincola	0.00	0.10	0.014	0.000	0.001	0.000	0.000	0.008
Oscillibacter	0.06	0.18	0.014	0.000	0.292	0.023	0.000	0.361
Oscillospira	0.02	0.12	0.010	0.000	0.966	0.257	0.000	0.966
Lachnospiraceae NK4B4 group	0.14	0.02	0.017	0.000	0.473	0.026	0.001	0.526
Peptococcus	0.13	0.34	0.029	0.000	0.003	0.008	0.002	0.020
Mitsuokella	6.41	0.27	0.809	0.000	0.041	0.042	0.003	0.100
[Eubacterium] eligens group	0.16	0.03	0.017	0.001	0.035	0.397	0.007	0.094
Collinsella	0.16	0.03	0.014	0.001	0.084	0.126	0.007	0.155
Ruminococcaceae UCG-005	0.96	2.51	0.179	0.001	0.038	0.551	0.008	0.097
Succinivibrio	1.42	0.13	0.177	0.001	0.018	0.434	0.008	0.058
Roseburia	4.00	1.47	0.324	0.001	0.000	0.099	0.010	0.002
Pseudoramibacter	0.11	0.00	0.016	0.002	0.096	0.405	0.015	0.165
Ruminococcus	2.58	1.49	0.170	0.003	0.125	0.000	0.018	0.193
Olsenella	0.82	0.04	0.147	0.004	0.036	0.053	0.023	0.094
Syntrophococcus	0.20	0.01	0.029	0.005	0.242	0.466	0.025	0.320
Butyricicoccus	0.16	0.04	0.014	0.007	0.809	0.151	0.031	0.830
Lachnospira	0.27	0.15	0.036	0.008	0.397	0.093	0.034	0.461
Lactobacillus	20.25	40.20	2.468	0.010	0.051	0.101	0.043	0.108
Dorea	0.57	0.27	0.042	0.011	0.094	0.392	0.044	0.165
[Eubacterium] nodatum group	0.17	0.05	0.029	0.017	0.016	0.973	0.061	0.056
[Eubacterium] ruminantium group	0.15	0.06	0.024	0.017	0.069	0.446	0.061	0.130
Desulfomonile	0.06	0.04	0.009	0.021	0.320	0.084	0.069	0.383
Faecalibacterium	4.55	1.54	0.421	0.020	0.050	0.917	0.069	0.108
Lachnospiraceae UCG-001	0.10	0.04	0.010	0.023	0.584	0.036	0.072	0.641
[Eubacterium] rectale group	1.02	0.46	0.101	0.033	0.001	0.132	0.090	0.010
[Eubacterium] xylanophilum group	0.52	0.20	0.055	0.031	0.278	0.003	0.090	0.354
Dialister	1.29	0.06	0.175	0.032	0.018	0.060	0.090	0.058
Treponema	1.01	2.03	0.254	0.031	0.009	0.108	0.090	0.036
Lachnospiraceae NK3A20 group	1.01	0.03	0.367	0.036	0.064	0.597	0.094	0.123
Oribacterium	0.30	0.17	0.023	0.041	0.243	0.294	0.101	0.320
Ruminococcaceae UCG-008	1.97	2.66	0.217	0.040	0.047	0.000	0.101	0.108
Prevotellaceae NK3B31 group	1.96	2.91	0.282	0.043	0.217	0.014	0.102	0.312
Ruminococcaceae UCG-009	0.04	0.07	0.006	0.045	0.434	0.151	0.105	0.490
[Ruminococcus] gauvreauii group	1.06	0.73	0.101	0.051	0.004	0.001	0.111	0.020
Rikenellaceae RC9 gut group	1.59	1.19	0.110	0.049	0.093	0.000	0.111	0.165

**Genus**	**Treatment**			**p-value**			**p-adjusted**	
	**Control**	**FB1**	**SEM**	**Group**	**Date**	**Group x Date**	**Group**	**Date**
Family XIII AD3011 group	0.31	0.65	0.060	0.053	0.000	0.019	0.113	0.005
Anaerostipes	0.50	0.79	0.141	0.055	0.236	0.166	0.114	0.320
Blautia	6.03	4.58	0.307	0.062	0.000	0.200	0.126	0.007
[Eubacterium] coprostanoligenes group	1.74	0.90	0.166	0.066	0.164	0.028	0.127	0.244
dgA-11 gut group	0.16	0.02	0.024	0.065	0.005	0.024	0.127	0.025
Bacteroides	0.10	0.13	0.034	0.068	0.109	0.605	0.128	0.180
Ruminiclostridium	0.42	0.55	0.031	0.083	0.232	0.085	0.152	0.320
Coprococcus	1.71	2.27	0.215	0.088	0.035	0.342	0.155	0.094
Sarcina	3.61	1.13	0.478	0.088	0.042	0.001	0.155	0.100
Family XIII UCG-001	0.08	0.06	0.006	0.097	0.014	0.011	0.167	0.052
Prevotellaceae UCG-003	0.19	0.08	0.024	0.117	0.004	0.001	0.196	0.021
Christensenellaceae R-7 group	1.49	2.55	0.361	0.121	0.001	0.003	0.199	0.008
Anaerotruncus	0.28	0.29	0.033	0.134	0.241	0.597	0.214	0.320
Fusicatenibacter	0.49	0.36	0.041	0.136	0.187	0.606	0.214	0.274
Odoribacter	0.15	0.11	0.064	0.138	0.013	0.326	0.214	0.051
Ruminococcaceae UCG-010	0.23	0.33	0.031	0.153	0.001	0.211	0.233	0.008
Ruminococcaceae NK4A214 group	0.49	0.71	0.066	0.211	0.003	0.004	0.315	0.020
Ruminococcaceae UCG-014	0.95	1.16	0.102	0.218	0.734	0.730	0.318	0.763
Parabacteroides	0.50	0.21	0.101	0.235	0.124	0.021	0.337	0.193
Subdoligranulum	1.02	1.57	0.160	0.265	0.049	0.872	0.373	0.108
[Eubacterium] hallii group	0.53	0.35	0.043	0.286	0.056	0.312	0.377	0.114
Anaerovibrio	0.07	0.13	0.015	0.281	0.632	0.445	0.377	0.675
Porphyromonas	0.22	0.09	0.064	0.273	0.311	0.776	0.377	0.378
Prevotella	8.30	6.52	0.612	0.286	0.060	0.685	0.377	0.119
Terrisporobacter	4.36	2.51	0.595	0.292	0.004	0.000	0.378	0.021
Lachnospiraceae ND3007 group	0.29	0.40	0.039	0.301	0.291	0.036	0.383	0.361
Intestinibacter	0.63	0.70	0.047	0.318	0.361	0.130	0.396	0.425
Ruminococcaceae UCG-013	0.09	0.11	0.012	0.321	0.015	0.056	0.396	0.052
Sphingomonas	0.26	0.22	0.018	0.325	0.156	0.593	0.396	0.237
Campylobacter	0.08	0.12	0.022	0.397	0.264	0.103	0.476	0.341
Acetitomaculum	0.10	0.10	0.024	0.549	0.685	0.341	0.647	0.721
Romboustia	0.08	0.33	0.093	0.557	0.020	0.167	0.647	0.060
Mogibacterium	0.14	0.15	0.012	0.610	0.052	0.019	0.698	0.108
Desulfovibrio	0.37	0.35	0.040	0.639	0.001	0.012	0.721	0.010
Alloprevotella	0.53	0.50	0.042	0.712	0.621	0.002	0.792	0.672
Lachnospiraceae NK4A136 group	1.16	1.14	0.116	0.743	0.008	0.085	0.815	0.033
Lachnoclostridium	0.53	0.58	0.128	0.800	0.020	0.386	0.832	0.060
Lachnospiraceae FCS020 group	0.13	0.14	0.010	0.796	0.922	0.331	0.832	0.933

**Genus**	**Treatment**			**p-value**			**p-adjusted**	
	**Control**	**FB1**	**SEM**	**Group**	**Date**	**Group x Date**	**Group**	**Date**
Lactococcus	0.10	0.04	0.047	0.771	0.119	0.104	0.832	0.193
Marvinbryantia	0.44	0.39	0.031	0.811	0.099	0.988	0.832	0.167
Ruminococcaceae UCG-002	1.25	1.14	0.119	0.795	0.001	0.019	0.832	0.008
Barnesiella	0.97	1.01	0.092	0.831	0.000	0.499	0.842	0.000
Clostridium sensu stricto	1.47	2.47	0.458	0.963	0.000	0.001	0.963	0.007

[1] FB1 pigs were fed with a diet supplemented with 12ppm/kg FB1 for 29 days; Control pigs received the same diet without any supplementation. Fecal sampling took place weekly.

[2] Values obtained with "false discovery rate" test.

Table S5. Diet composition in percentage (%).

**Ingredients**	**%**
Wheat	47,500
Soya meal	24,300
Barley	22,900
Sunflower oil	1,400
Phytase	0,010
Lysine	0,465
DL-Methionine	0,165
L-Threonine	0,195
L-Tryptophan	0,045
Salt	0,235
$CaHPO_4$	1,120
Calcium carbonate	1,000
Premix[1]	0,665

1 Composition per kg of premix 2 000 000 UI of vitamin A, 400 000 of vitamin D3, 4 000 mg of vitamin E, 8 000 mg of vitamin C, 400 mg of vitamin B1, 1000 mg of vitamin B2, 2 170 mg of Pantothenic acid calcium salt, 4 000 mg of niacin, 6 mg of vitamin B12, 1000mg of vitamin B6, 400 mg of vitamine K3, 200 mg of folic acid, 40 mg of biotin, 12000 mg of choline chloride, 20 750 mg of Fe, 4 000 mg of Cu, 19 860 mg of Zn, 8 000 mg of Mn, 120 mg of I, 200 mg Co, 60 mg of Se.

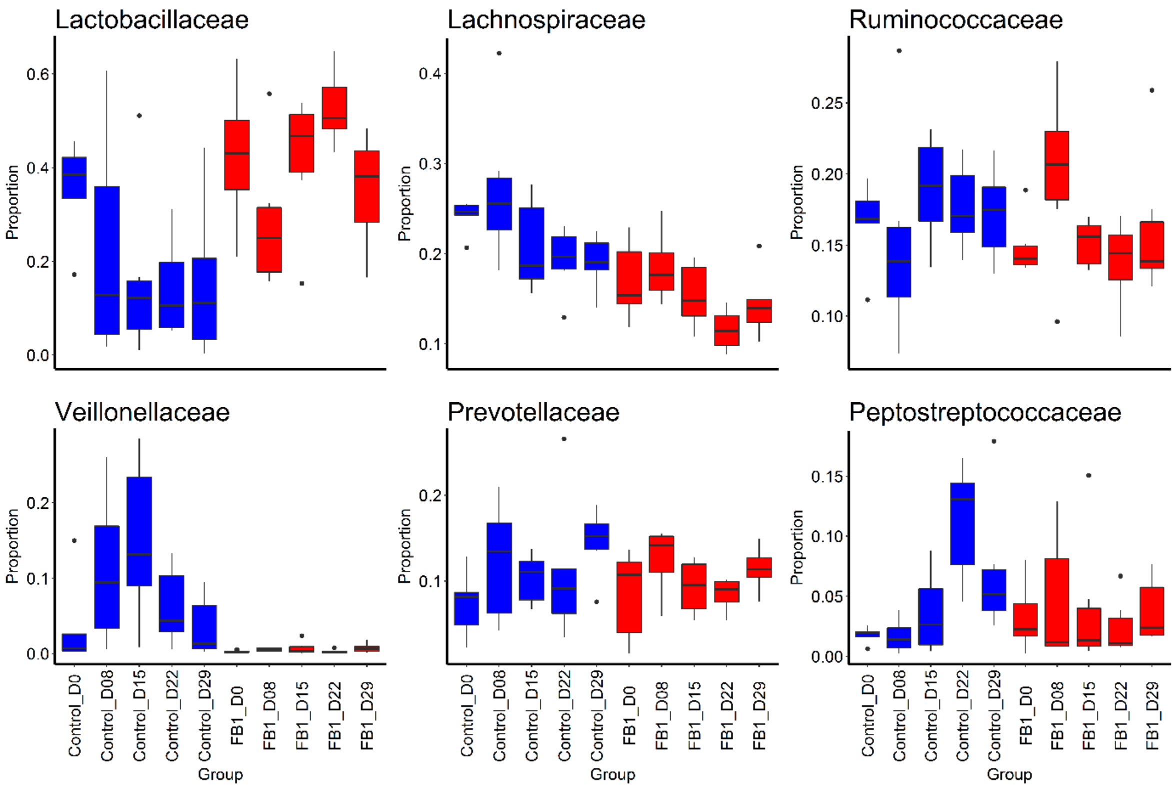

Figure S1. Relative abundance of main bacterial families in fecal microbiota from Control (blue) vs. FB1-exposed pigs (red).

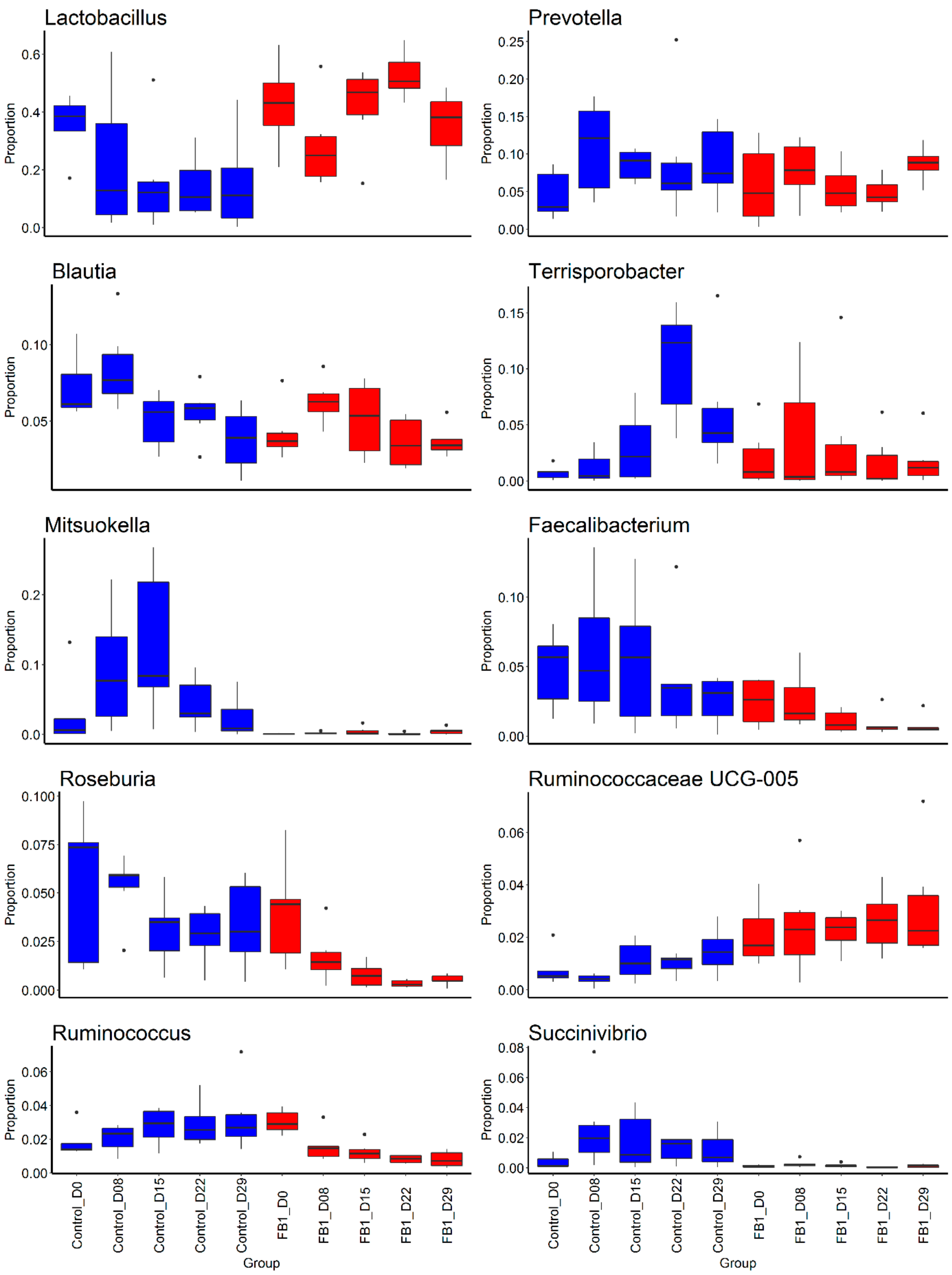

Figure S2. Relative abundance of main bacterial genera in fecal microbiota from Control (blue) vs. FB1-exposed pigs (red).

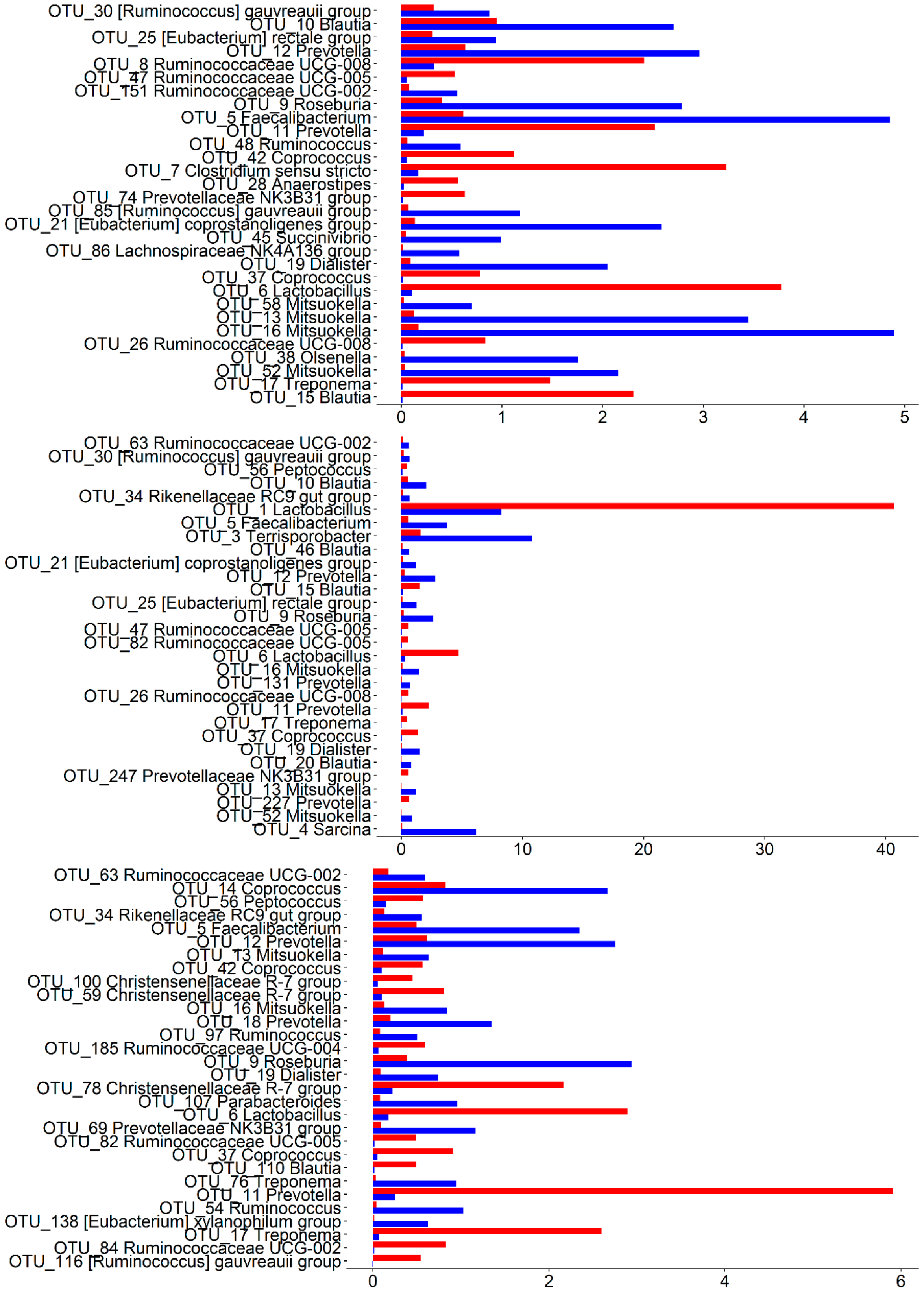

Figure S3. Relative abundance of 30 most abundant OTUs differentially abundant in fecal microbiota from Control (blue) vs. FB1-exposed pigs (red) at day 15 (top) day 22 (center) and day 29 (bottom) after FB1 exposure.
